# Active Commuting as a Factor of Cardiovascular Disease Prevention: A Systematic Review with Meta-Analysis

**DOI:** 10.3390/jfmk9030125

**Published:** 2024-07-18

**Authors:** Claudia Baran, Shanice Belgacem, Mathilde Paillet, Raphael Martins de Abreu, Francisco Xavier de Araujo, Roberto Meroni, Camilo Corbellini

**Affiliations:** 1 Department of Physiotherapy, LUNEX University of Applied Sciences, 4671 Differdange, Luxembourg; claudiabaran@live.fr (C.B.); belgshanice@gmail.com (S.B.); khloe.pai@gmail.com (M.P.); 2 Department of Physiotherapy, LUNEX ASBL, Luxembourg Health & Sport Sciences Research Institute, LUNEX University of Applied Sciences, 4671 Differdange, Luxembourg; rmeroni@lunex.lu (R.M.); ccorbellini@lunex.lu (C.C.); 3 Department of Gymnastics and Health, Federal University of Pelotas, Pelotas 96010-610, Brazil; francisco.araujo@ufpel.edu.br

**Keywords:** cardiovascular disease, prevention, commuting

## Abstract

Active commuting (AC) may have the potential to prevent the incidence of cardiovascular disease (CVD). However, the evidence for a correlation between AC and the risk of CVD remains uncertain. The current study thoroughly and qualitatively summarized research on the relationship between AC and the risk of CVD disease. From conception through December 2022, researchers explored four databases (PubMed, PEDro, Cochrane, and Bibliothèque Nationale of Luxembourg [BnL]) for observational studies. The initial findings of the search yielded 1042 references. This systematic review includes five papers with 491,352 participants between 16 and 85 years old, with 5 to 20 years of follow-up period. The exposure variable was the mode of transportation used to commute on a typical day (walking, cycling, mixed mode, driving, or taking public transportation). The primary outcome measures were incident CVD, fatal and non-fatal (e.g., ischemic heart disease (IHD), ischemic stroke (IS), hemorrhagic stroke (HS) events, and coronary heart disease (CHD). Despite methodological variability, the current evidence supports AC as a preventive measure for the development of CVD. Future research is needed to standardize methodologies and promote policies for public health and environmental sustainability.

## 1. Introduction

According to the World Health Organization (WHO), cardiovascular diseases (CVD) are the leading cause of disability and death worldwide, with 17.9 million deaths per year [1]. Coronary heart disease (CHD), cerebrovascular disease, rheumatic heart disease, and other illnesses are among the category of heart and vascular disorders known as CVD [2]. The American Society for Preventive Cardiology (ASPC) states that CVD risk factors include unhealthy eating, inactivity, dyslipidemia, pre-diabetes/diabetes, elevated blood pressure, obesity, usage of tobacco products, alcohol abuse, and family history/genetics/familial hypercholesterolemia [3]. Therapeutic lifestyle modifications such as increased physical activity (PA), dietary modification, weight loss, and smoking cessation are beneficial in reducing the risk of CVD [4]. The WHO advises 150 min of moderate PA per week for significant health advantages. However, most adults in high-income nations do not engage in the recommended level of PA and instead spend most of their waking hours sitting down [5,6]. Several studies report that this behavior raises morbidity and mortality risks from most non-communicable diseases, including CVD [7,8].

Bassett et al. [9] claimed that in countries where active travel is decreasing, obesity rates are increasing. Percentage of body fat, body mass index (BMI), waist circumference, mental health and physical well-being is impacted by daily walking or bicycling [10,11,12,13]. Further, disorders like diabetes or arterial hypertension are less common in pedestrians and cyclists and have a lower risk of CHD than commuters who use cars [12,13,14,15,16,17]. Additionally, studies revealed that frequent cycling reduces mortality from all causes by about 30% [18,19]. PA has important mechanisms for blood pressure control, lipid-lowering, anti-inflammatory effects, improved endothelial function, and glycemic control [16]. However, lack of time is commonly mentioned in the literature as a significant obstacle to raising daily PA levels in industrialized countries [20,21,22,23,24,25,26,27].

Other than participating in sports, various strategies exist for increasing PA levels; one is active commuting (AC). It refers to how people travel daily from home to work, school, or hobbies. More specifically, travel is possible solely by walking, bicycle use, or in conjunction with motorized transportation modes, for example, by combining driving and walking [28]. This form of PA may be included in daily living, considering the increase in sedentary lifestyles [2,29]. AC may potentially decrease CVD risk by increasing PA [30]. Indeed, there are various types of AC, but walking and biking are the most common. Mixed commuting occurs when a person uses multiple forms of transportation to travel to a place. It is conceivable, for example, to ride a bike to the train station and then take the train to work. It can be an excellent method of exercise while also traveling to a specific location. Each has its own set of advantages, such as reducing cardiovascular risk, ameliorating rising health-care costs, and minimizing environmental impact by lowering carbon emissions, with only a modest increase in the number of activity-related injuries [30]. Additionally, these have been reported across all age groups, including lower risk of developing coronary heart disease, better physical function and mental health in middle-age and older adults, and enhanced fitness, mental well-being, and academic performance in younger populations [30].

Most studies focused on PA to prevent CVD, and fewer reported the independent effects of walking or cycling from commuting, meaning results may be confounded by other PA [31,32,33]. People who walk or cycle more frequently may engage in more leisure time or work-related PA, inflating the health advantages of these behaviors. The majority of the research reported that AC minimizes obesity and overweight [34]. Although some extensive cohort studies demonstrated preventive effects concerning various health outcomes (including CVD), others reveal no effects of AC [15,35,36]. Active transportation, more PA, and lower body weight were significantly correlated in three recent systematic analyses investigating the connection between AC, PA level, body weight, and health outcomes in adults [14,34,37].

However, only a few studies have established a direct link between AC as a primary factor and CVD prevention, which limits the understanding of the impact of this approach on cardiovascular health. Therefore, conducting a systematic review is essential for clarifying the diverse health effects of AC behaviors on CVD prevention. The results of this study will provide a clear, evidence-based foundation for public health strategies and interventions by addressing existing gaps in the literature and offering a solid basis for promoting physical activity as a key preventive measure against CVD. To address this gap, this study aimed to systematically review the literature to examine the association between AC and CVD prevention.

## 2. Materials and Methods

We conducted a systematic review including collected and analyzed analytical data from several electronic databases according to the PRISMA (Preferred Reporting Items for Systematic Reviews and Meta-Analyses) [38]. The protocol is registered in the International Prospective Register of Systematic Reviews (PROSPERO) under the following registration number: CRD42023391453.

### 2.1. SPIDER

For a well-focused research question to improve the efficiency of the literature search, the present review followed the SPIDER framework (Sample/Phenomenon of Interest/Design/Evaluation/Research type) [39]. This systematic review selected a population of healthy participants older than 16 years old at baseline who commute actively.

### 2.2. Eligibility Criteria

Criteria for inclusion were (1) participants older than 16 years old; (2) prospective cohort, case-controlled study or cross-sectional study; (3) English language publication; (4) examining the relationship between the cardiovascular outcome (cardiovascular mortality, incident CHD, stroke) and the type of commuting (active/non-active); and (5) publication published between 2015 and 2022. Prospective studies on non-healthy participants at baseline, with less than 5-year follow-up period, and other diseases such as cancer are excluded.

### 2.3. Outcome Measures

The following outcomes were selected as primary outcomes: CVD, fatal and non-fatal (e.g., IHD, IS, HS events, CHD) and as secondary outcomes: BMI, systolic/diastolic blood pressure, waist and hip circumference, VO2max, hypercholesterolemia, diabetes.

### 2.4. Data Sources and Search Strategy

A search was performed in PubMed, PEDro, Cochrane and the BnL to find relevant publications. The following Mesh terms were the keywords used in the search: “commuting” and “cardiovascular diseases”. Additionally, various commuting strategies and symptoms of cardiovascular conditions or disease synonyms were established as secondary terms (Table 1).

To identify as many pertinent articles as possible, a combination of these keywords was applied to the databases using Boolean operators, which include “AND” and “OR.” Filters were applied: “humans”, “7 years”, “English”, and “clinical trial”. Up to December 2022, all significant papers published were included in the manually selected databases. Different combinations were verified to build a reliable search strategy and obtain complete articles relevant to the topic. Several research syntaxes were employed to create an effective search strategy since each databank’s guidelines have different properties. While the search string in PubMed goes with the following terms: #1 (commuting [Title/Abstract]) OR (active travel [Title/Abstract]) OR (active transportation [Title/Abstract]) OR (active transport [Title/Abstract]) OR (active commuting [Title/Abstract]) OR (walking [Title/Abstract])) OR (bicycling [Title/Abstract]) OR (cycling [Title/Abstract]), #2 (“rheumatic heart disease” [MeSH Terms] OR “rheumatic heart disease” [MeSH Terms] OR “rheumatic heart disease” [MeSH Terms], #3 (“stroke” [MeSH Terms] OR “stroke” [MeSH Terms] OR “stroke” [MeSH Terms] OR “stroke” [MeSH Terms] OR “stroke” [MeSH Terms] OR “cerebrovascular disease” [All Fields], #4 (“cardiovascular diseases” [MeSH Terms] OR (“cardiovascular” [All Fields] AND “diseases” [All Fields]) OR “cardiovascular diseases” [All Fields] OR (“cardiovascular” [All Fields] AND “disease” [All Fields]) OR “cardiovascular disease” [All Fields]) OR (“cardiovascular diseases” [MeSH Terms]) OR “cardiovascular disease incidence” [All Fields] OR “cardiovascular disease mortality” [All Fields] OR “CVD” [All Fields], #5 “coronary disease” [MeSH Terms] OR (“coronary” [All Fields] AND “disease” [All Fields]) OR “coronary disease” [All Fields] OR (“coronary” [All Fields] AND “heart” [All Fields] AND “disease” [All Fields]) OR “coronary heart disease” [All Fields]) OR “coronary disease” [MeSH Terms] OR “acute coronary syndrome” [MeSH Terms] OR “coronary disease” [MeSH Terms] OR “coronary disease” [MeSH Terms], #6 (#2 OR #3 OR #4 OR #5),#1 AND #6. The Cochrane Library (*n* = 14), the BnL (*n* = 73) and PEDro (*n* = 2) use other keywords like “active commuting” AND “cardiovascular disease” and “Active” AND “Commuting” AND “Cardiovascular disease”.

### 2.5. Study Selection

Title and abstract scanning were completed independently by three group members (SB, CB, MP) while considering the topic and possible inclusion. The full-text screening was performed where it was unclear whether the article should be included in the title and abstract. All the selected databases offered all researchers unrestricted access, except for the article of Peterman et al. [40]. The researchers debated and decided on the article selection before performing a second independent analysis on the chosen complete texts in light of the previously determined inclusion/exclusion criteria. Any discrepancies were resolved via consensus. All team members had a discussion and approved the final research inclusion. Furthermore, the methodological quality of every included study was assessed and reviewed.

### 2.6. Quality Assessment

Three reviewers (MP, CB, SB) independently assessed each included study’s internal validity and methodological quality (Table 2), and disagreements were resolved via consensus. The National Institutes of Health (NIH) scale was used to evaluate the research quality for observational cohort and cross-sectional studies [41]. The internal validity was examined using the Evaluation of Public Health Practice Projects (EPHPP) Quality Assessment tool to evaluate the risk of bias (RoB) [42]. EPHPP analyzes the internal validity of the studies, which presents six domains: selection bias, study design, confounders, blinding, data collection method, withdrawal and dropout [42].

### 2.7. Data Extraction

By using a standardized form, all data were extracted by two reviewers (MP, CB). Disagreements were resolved with the opinion of a third investigator (S.B.). The complete data extraction for each included study is provided in Table 3.

The data extracted were lead author and year of publication, country of the study population, data sources, study design, sample size, age of participants, length of follow-up (years), exposure, exposure measurement, other PA domains assessed, the definition of the outcome, number of events and details of adjustment for confounding factors in the multivariable model.

### 2.8. Statistical Analysis

The change in CVD, fatal and non-fatal (e.g., IHD, IS, HS events, CHD) BMI, systolic/diastolic blood pressure, waist and hip circumference, VO2max, hypercholesterolemia, and diabetes were used for analysis. When not provided, the standard deviation (SD) of changes was calculated using a correlation coefficient, according to the recommendations of the Cochrane Handbook for Systematic Reviews of Interventions. All meta-analyses were performed using the Review Manager Software version 5.3 (Copenhagen: The Nordic Cochrane Centre, The Cochrane Collaboration, 2014), and we used a random-effects model for both meta-analyses.

### 2.9. Certainty of Evidence

Following the Grading of Recommendations, Assessment, Development, and Evaluation (GRADE) device (Table 4), different degrees of evidence distinguished the certainty of the evidence analysis, which has five domains: limitations in study design or execution (RoB), inconsistency of results, indirectness of evidence, imprecision, and publication bias [48]. The evidence was divided into four levels: high quality (all five domains are satisfied), moderate quality (one of the five domains is not satisfied), low quality (two of the five domains are not satisfied), and very low quality (three of five domains are not satisfied) [49].

If there was an unclear or high RoB and significant limitations in the estimation effect, recommendations for the RoB domain were lowered. The inconsistency category was downgraded when the results between studies were not coherent. They were downgraded when substantial differences were found in interventions, demographics, or outcomes for the indirectness domain. The imprecision category was downgraded if the sample size was not significant. Finally, if publication bias had a significant influence, it was downgraded.

## 3. Results

### 3.1. Study Characteristics

**Figure 1:**
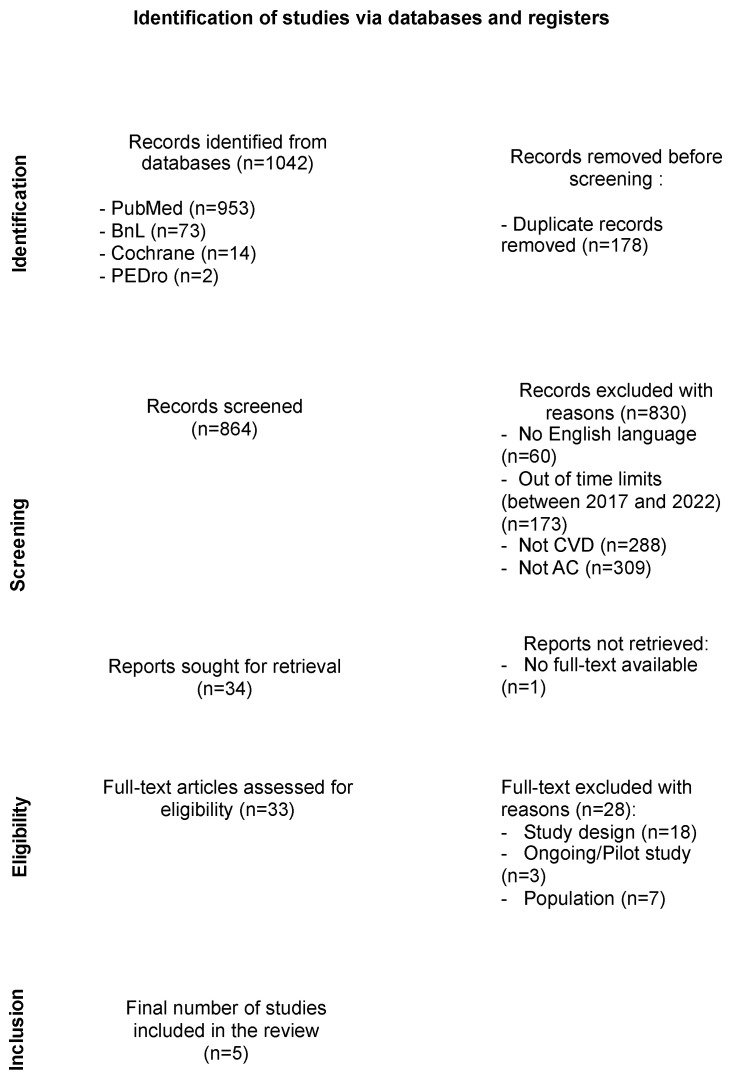
PRISMA flowchart.

The search yielded 1042 records: 953 from PubMed, 73 from BnL, 14 from Cochrane, and 2 from PEDro. Duplicates were removed, leaving 864 records. After screening titles and abstracts, 33 records were selected for full-text evaluation following the exclusion and inclusion criteria. The PRISMA flow diagram (Figure 1) summarizes the steps followed to reach the five articles selected after the full-text reading. Thirty-three full-text articles were selected for the eligibility phase. After thoroughly reading the picked articles, 28 were excluded for different reasons. Consequently, these rejected studies indicate characteristics that did not correlate to the project; 18 presented a different study design than those considered (e.g., randomized control study or systematic review), and 3 others were ongoing studies that were therefore not completed or pilot studies. In the last seven removed studies, the population encompassed participants under 16 (Figure 1).

Five studies, involving 491.352 participants, were included. The participant age ranged from 16 to 85. The follow-up period ranged from 5 to 20 years (Table 3). Studies were undertaken in Asia [46], the United States [47] and Northern Europe [43,44,45]. Overall, mixed-mode: cycling and walking commuting were analyzed in four studies, and cycle commuting was investigated in one study [44]. All studies used different data sources (Table 3).

### 3.2. Methodological Quality and Risk of Bias

**Figure 2:**
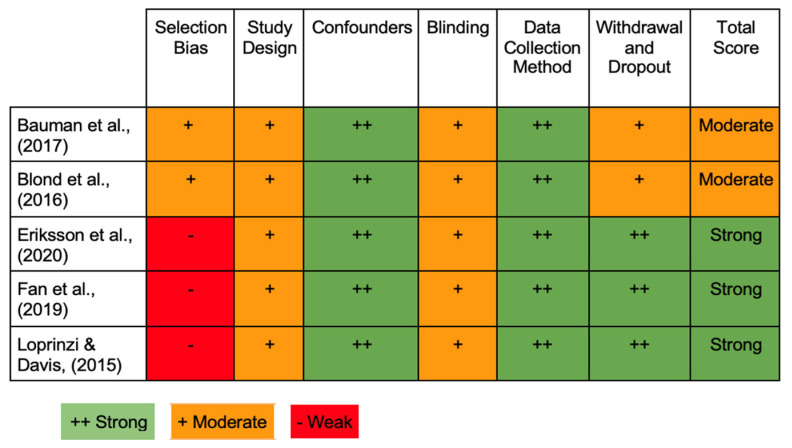
The evaluation regarding the internal validity in the included studies (EPHPP) [43,44,45,46,47].

The five studies involved in this research were of high quality, as reported in Table 2. Bauman et al. [43] were rated 11/1447 on the NIH tool, while Blond et al. [44], Eriksson et al. [45], and Fan et al. [46] obtained a score of 12/14 [44,45,46], and Loprinzi and Davis [47] obtained the highest score [47], 13/14. Figure 2 displays the RoB of each included study. Internal validity was classified as weak, moderate, or strong for each domain. The studies by Fan et al. [46] and Loprinzi and Davis [47] were assessed as strong quality [46,47], whereas the other three studies were rated as moderate quality [43,44,45]. The inter-examiner (CB, SB, MP) reliability revealed a high agreement level.

For the five different articles, the following results regarding the six primary domains of the EPHPP were reported:

- Selection bias: three papers were determined to have a high RoB [45,46,47], while two studies were deemed to have a moderate RoB [43,44].
- Confounding bias: the five articles were given a low RoB rating [43,44,45,46,47].
- Detection and performance bias: all the studies were not double-blinded. Thus, they were given a moderate RoB [43,44,45,46,47].
- Attrition bias: three studies were identified with a low level of bias [45,46,47]. Bauman et al. [43] and Blond et al. [44] revealed a moderate bias level due to missing information concerning the dropout [43,44].
- Reporting bias: all five articles were classified with a low-level bias in this domain.
- Even though the RoB ranged from high to low, two studies rated a moderate RoB [43,44], and the three remaining studies provided a high RoB [45,46,47].

### 3.3. Certainty of Evidence

The certainty of evidence regarding the effects of AC on CVD prevention are reported in Table 4. The certainty of evidence strongly supports AC’s performance in CVD prevention. Regarding the reliability of the results, all of the researchers (CB, SB, MP) indicated a high level of agreement.

### 3.4. Effect of AC on CVD

AC exposure levels were reported as minutes spent walking or cycling for transportation per day, as dichotomized variables (“yes” or “no” for any active transportation), or as metabolic equivalent of tasks, indicating a lack of consistency in the definition of AC across studies. The reference category was reported in most studies as the absence of AC. Three studies [44,45,46] examined the correlation between AC and CVD incidence (including IHD disease, stroke, HS, angina pectoris, and non-fatal myocardial infarction) (*n* = 16,586). AC was associated with a considerably decreased risk of CVD occurrence. Three studies (*n* = 646) investigated the link between AC and CVD mortality [43,45,47]. All the databases used the International Classification of Diseases (ICD) for reporting cases of CVD events, death caused by CVD and death from CHD: the 8th revision [43,45], the 9th revision [45], the 10th revision [43,44,45,46,47], the 8th and the 10th revision [43] or the 8th, 9th, and 10th revisions [45].

Bauman et al. [43] published a cross-sectional study to investigate whether ‘PA patterns’ (consistently low, consistently high, or inconsistent PA levels across time) had different epidemiological correlations for anthropometric, mortality, and CVD/CHD outcomes than a single time-point assessment of PA. Participants (*n* = 4581) were mainly men (51.1%), healthy at baseline, and referred to a 19-year outpatient national study. They were examined through three waves 1982/1983 (time 1), 1987/1988 (time 2) and 1993/1994 (time 3), with a dropout rate of 36%. At the baseline and times 2 and 3, the examinations were assessed with a questionnaire based on Saltin and Grinby’s questionnaire [50]. Participants were divided into different groups related to their level of AC: low (0–19 min/day), moderate (20–39 min/day), and high (40+ min/day). Subjects were also divided into three levels depending on their PA in leisure time (level 1: none, level 2: moderate <4 h, level 3: moderate or strenuous >4 h). Participants in this study were also assessed for covariates (weight, height, waist, and hip circumference). Cox proportional hazard models and competing risk analyses were applied to examine correlations with CVD/CHD cause-specific mortality. The results were shown as hazard ratios (HR) and sub-hazard ratios (SHR), together with 95% confidence intervals (CI), for the relevant category in comparison to the reference category. Stata 13.0 was used for all analyses, and the significance level was set at *p* < 0.05.51. In the strenuous group at time 3, there was a 43% risk reduction in CVD deaths (HR 0.57; 95% CI 0.35–0.93). The HR for active commuters was similar (HR 0.61; 95% CI 0.33–1.15), but it did not reach statistical significance (*p* < 0.05). Subjects who commuted for >4 h per week revealed a risk reduction of 62% (HR 0.38; 95% CI 0.15–0.96) and none of the single time point measures. The numbers of CHD (*n* = 69) and CVD death-related (*n* = 185) were low. These numbers contributed to low statistical significance despite the expected trend.

Blond et al. [44] conducted a prospective study of Danish men and women to examine the connection between cycling, changes in cycling habits, and the risk of CHD incidents. From 1993 to 1997, 53,723 Danes (25,329 men and 28,394 women) from 50 to 65 years old were recruited from the prospective cohort study “Diet, Cancer, and Health” over 20 years. A self-reported questionnaire assessed overall cycling (commuter or leisure-time cycling) and covariates at baseline and in a second experiment (from 1999 to 2003). Participants who lost their jobs or retired during the follow-up were no longer considered commuters. Participants with a history of stroke, CHD, or cancer were excluded at baseline. In total, 23,283 participants dropped out (43%) during the study. CHD risk was estimated using Cox proportional hazards regression in connection to weekly cycling duration categories and changes in cycling behaviors from baseline to second examination. There were 2892 incident instances of CHD detected during 20 years of follow-up. In a multivariable-adjusted examination of overall cycling, cyclists had an 11% to 18% reduced risk of CHD compared to non-cyclists. Changing cycling habits from no cycling to cycling between baseline and second examination was linked with a 26% decreased CHD risk relative to no cycling. An estimated 7.4% (HR 0.926; 95% CI 3.6–11.1) of all CHD cases may be avoided if all individuals in this sample cycled or continued to cycle for leisure or commuting activities.

Eriksson et al. [45] aimed to describe trends in AC between 1998 and 2015 and to investigate the relationship between various levels of AC and the incidence risk of CVD in a large sample of men and women from the Swedish working population. Participants in this study were also assessed for adjustment variables (Table 3). The data were from 318.309 healthy participants (47% women) aged 18–74 years. A total of 27,017 people dropped out due to not answering the relevant question about physical working conditions. In total, 291,292 people were registered to the physical work condition subgroup. Participants could travel by car, bus, rail, walking, or cycling. The level of AC (walk or cycle) was reported by groups of duration in min/day, <10 min, between 10 and 19 min, between 20 and 29 min, or >30 min. Participants who identified their transportation method as either by vehicle, bus, or train or 5 min/day were classified as “passive commuters” in the descriptions of the trends in AC and the association with CVD risk. One-way ANOVA and Kruskal–Wallis ANOVA were used to examine differences among the various commuter groups, with post hoc analysis to account for multiple comparisons. The prevalence of first-time CVD and baseline commuting behaviors were compared using Cox proportional hazard regression modeling to calculate HR and 95% CI. Results were unaltered by sensitivity analyses that included all CVD incidence rates (*n* = 5714) and incidence rates that excluded any incidents within the first two years (*n* = 4640). Scaled Schönfelts residuals were used to test the proportionality assumption for Cox regression, finding no evidence of a breach. IBM SPSS52 was used to analyze the data. A total of 5714 first-time CVD incidents occurred over a mean follow-up period of 7.2 years. Compared to passive commuters, low-dose active commuters at baseline had a risk reduction of 11% (HR 0.89; 95% CI 0.83–0.95), and moderate/high-dose active commuters at baseline had a risk reduction of 9% (HR 0.91, 95% CI 0.83–0.98) and had a significantly lower risk (*p* < 0.05) of a first-time CVD event after multi-adjustment for covariates without the ability to further differentiate between the modes of transportation (walking or cycling), the intensity of the commute, or the distance covered.

Fan et al. [46] investigated the relationship between AC and the risk of incident CVD. A total of 104,170 (48.6% female) urban commuters without severe chronic illnesses with a mean age of 45.9 years old at baseline were provided. Non-AC subjects work at home or near home. Walking and cycling were self-reported commuting types. Participants who reported not working (*n* = 110,670), a history of heart disease (*n* = 10,453), stroke (*n* = 5241), or cancer (*n* = 1390) were excluded. Those who were recorded with an unlikely censoring date for loss to follow-up (*n* = 1) were excluded. The incident risks of major CVD related to baseline commuting mode were estimated using stratified Cox regression, with stratification on age at risk (5-year intervals) and study area. All comparisons were performed based on the two-tailed test, and the significance level was set at *p* < 0.05. The level of PA was described by groups of both intensity and duration (MET-hours/days). The commuting behavior was self-reported thanks to a questionnaire designed for the study, and the daily commuting time (min) was recorded. In the study, four commuting categories were described—1: non-active (motorcycle, by car, or by bus/ferry/train); 2: mainly work at home or near home; 3: walking; 4: cycling. The level of AC for walking and cycling was self-reported by groups of duration in min/day: <15, 15 to 29, 30 to 59, and ≥60 min/day. The researchers collected covariates in the baseline questionnaire (Table 3). In all, 20.1% of participants reported walking, 19.4% reported cycling, 13.4% stated working from home or close to home, and 47.2% reported non-AC: except for a family history of heart attack (*p* = 0.035), family history of stroke (*p* = 0.845), and hypertension (*p* = 0.443), all *p*-values for the trend were less than 0.003. There were significant dose-response trends for IHD incidence by daily commute time among active commuters (*p* = 0.001 for trend). After covariate adjustment (Table 3), reduced risk of developing IHD by 10% for working at home or near home (HR: 0.90; 95% CI 0.82–0.99), 10% for walking (HR, 0.90; 95% CI 0.84–0.96), and 19% for cycling (HR: 0.81; 95% CI 0.74–0.88) than non-AC. There was no significant association (*p* > 0.05) for work at/near home, walking, or cycling for HS and IS. The risk reduction for IHD for those who reported walking 15, 16 to 29, 30 to 59, and 60 min were 0% (HR: 1.00; 95% CI 0.87–1.15), 5% (HR: 0.95; 95% CI 0.85–1.06), 13% (HR: 0.87; 95% CI 0.79–0.95), and 18% (HR: 0.82; 95% CI 0.71–0.95), respectively, compared to those who reported non-AC. The risk reduction for IHD for those who reported cycling 15, 16 to 29, 30 to 59, and 60 min were 15% (HR: 0.85; 95% CI 0.68–1.07), 27% (HR: 0.73; 95% CI 0.63–0.86), 18% (HR: 0.82, 95% CI 0.73–0.92), and 21% (HR: 0.79; 95% CI 0.67–0.92). HS and IS incidence by daily active commute time showed no dose-response tendencies (*p* > 0.05).

Loprinzi and Davis [47] conducted a study investigating the individual, combined, and isolated effects of movement-based behaviors (MBB) on CVD-specific mortality. The analyses were based on information supplied by 15,327 persons (20–85 years old) with a mean age of 45 and 51.3% of women who submitted complete data for the research variables. It eliminated individuals with incomplete MBB data and lacking covariate data; there were 12,339 remaining. Finally, 12,321 people remained after excluding those with missing mortality status or period to follow-up, making up the analytic sample. The number of dropouts was 18. This study did not differentiate active transport (walking, cycling, and public transportation) and was investigated as AC. The MBB was self-reported with a questionnaire specifically designed for the study into four items: moderate-intensity aerobic PA (MPA), vigorous-intensity aerobic PA (VPA), muscular strength activities (MSA), and active transport (yes/no responses for each MBB). Stata techniques were used to conduct statistical analyses on survey data (v.12). To investigate the relationship between MBB and CVD-related mortality, Cox proportional hazard models were applied. The proportional hazard assumption was established using Schoenfeld’s residuals. Six hundred and fifty-four participants died from all causes over the five-year follow-up period (5 years), with 231 deaths attributable to CVD. The MBB log-rank test for equality for CVD-specific mortality also showed significance (chi-square = 57.27; *p* < 0.001). Regular PA involvement was linked to lower all-cause and CVD-specific mortality, according to studies. After covariate adjustment (Table 3), the authors examined the independent effects of each MBB, and they presented a reduced risk of CVD mortality of 66% for VPA (HR: 0.34; 95% CI 0.19–0.60), 49% for MPA (HR: 0.51; 95% CI 0.32–0.83) and 29% for active transport (HR: 0.71; 95% CI 0.41–1.23). And after examining MBB in isolation on CVD mortality, they presented a reduction in CVD mortality of 56% for VPA (HR: 0.44; 95% CI 0.19–1.05), 36% for MPA (HR: 0.64; 95% CI 0.38–1.08), 40% for active transport (HR: 0.60; 95% CI 0.31–1.16). The results were only significant (*p* < 0.05) for MPA and VPA. They were inversely associated with CVD-specific mortality, with VPA more strongly associated with reduced CVD mortality risk than MPA. No significant individual effects for MSA or active transport on mortality risk were observed (*p* > 0.05). The researchers suggested that it was related to the non-differentiation between the different types of commuting.

## 4. Discussion

### 4.1. Summary of Main Findings

This systematic review examined AC’s impact on CVD prevention in healthy individuals. We found that daily AC positively benefits CVD prevention with a decreased risk of CVD. The more understanding of the association between AC and CVD incidence/mortality, the more robust the recommendations should be that are made to enhance public health. Consistently walking and cycling were related to lower risks of having CVD, especially for individuals who commuted for longer periods.

### 4.2. Strengths and Limitations

This systematic review has been performed following a comprehensive search in electronic databases. We conducted this review according to the PRISMA guidelines, critically appraised the included studies and determined the certainty of evidence using the GRADE framework. Findings should be interpreted with caution because the included research presents significant limitations. In most studies, mostly men were present in samples, except for Loprinzi and Davis [47] and Blond et al. [44], who reported mostly women [44,47]. Additionally, the included studies utilized various control and adjustment variables, potentially affecting their homogeneity. These differences can lead to variations in outcomes, making it challenging to draw consistent conclusions and affecting the reliability and comparability of the results. The key consideration was analyzing the influence of AC on CVD prevention, which was another restriction in this analysis: none of the five studies specifically explored the influence of AC on CVD employing the same outcome measure, and AC was not applied with the same established protocol. Moreover, the presented data’s quality was limited because using PA self-report assessments is known to underestimate risk, and a consistent behavioral pattern may more accurately reflect real exposure. Across studies, the definition of AC varied. For instance, failing to distinguish between the various forms of public transportation used and putting in the public transportation group those who reported using a combination of motorized and active transportation might produce unreliable findings. Besides the various periods of AC, the duration varied from study to study, emphasizing the lack of a procedure for structuring effective AC. The duration of AC was missing in the study of Loprinzi and Davis [47], while all of the other studies demonstrated a heterogeneous AC duration: duration per day [45] or week [43,44], MET-hour/days [46]. The speed of AC was not detailed in any study. The AC mode is relevant as cycling and walking may differ in intensity, which may improve CVD biomarkers differently [50]. Although measuring VO2max provides insights into exercise intensity, the dose–response relationship for cardiovascular fitness, long-term health benefits, and the comparative effectiveness of different commuting modalities in CVD prevention, recording heart rate during exercise and completing VO2max tests were deemed unfeasible for such large-scale cohort studies. A careful examination of intensity should still be carried out. Both low and moderate/high dose AC had comparable considerably lower risks for a first CVD during follow-up than passive commuters. There were no studies on eliminating PA during the experience and only using AC. Additionally, some studies considered subjects’ PA without quantifying it. Another limitation is that no meta-analysis was carried out on the results. The data and clinical heterogeneity from the study outcomes are the main reasons to avoid such analysis, as it would increase the bias of presenting inconsistent conclusions regarding the effect of AC on CVD [51].

### 4.3. Comparisons across Studies

Overall, there was some concordance and consistency in the content and modality between the studies. Furthermore, the large sample size, in addition to the long-term follow-up, was one of the study’s main strengths. The relationships between AC and major CVDs were consistent across all levels of PA in other domains. Despite the significant restrictions, all of the investigations indicated that AC enhanced CVD prevention. The positive effect of leisure time PA on all-cause and cardiovascular mortality was largely demonstrated with conflicting results for AC to and from work [52,53,54]. Commuting time and distance between home and work vary by country, and this time influences how people commute. Workers in European Union countries spent an average of 25 min traveling to work in 2019 [55]. Some factors to consider for AC options are rush-hour traffic, transportation/gas prices, and weather issues. As stated by Jones and Ogilvie [28], PA was not a main motivation when choosing modes of transport for daily travel, but convenience, speed, cost, and reliability were. The results indicated that more efforts should be made to develop plans to enhance AC behaviors. AC should be encouraged as an ecological goal in addition to its health advantages [55,56]. Global warming may be somewhat mitigated by AC [57]. A total of 120 to 130 g of CO_2_ is released into the environment per kilometer at a typical gasoline consumption rate of 5 L per 100 km [57]. Considering the number of workers using a car for less than 5 km commuting to and from work, this practice can produce billions of tons of CO_2_ emitted per year [58]. Policymakers must focus on finding a way to include AC in urban life [59]. Numerous strategies to promote AC should be put in place, including infrastructural improvements to make roads safer, extensions of bike networks, and financial incentives for behavioral change. Given that most workers commute by a short-distance car ride, AC might represent a significant change with considerable public health advantages [58,60,61]. It is important to comment that these advantages may be limited to pollution exposition, as a previous prospective study demonstrated an increased risk of CVD when the commuter is exposed to high concentrations of PM [25,61].

### 4.4. Future Perspectives

Future research should aim to provide a clearer and more standardized description of active AC, including its type, duration, intensity, and frequency. This would involve developing consistent methodologies for measuring AC and focusing on the quality of life (QoL) of participants. High-quality clinical trials designed specifically to measure the effect of AC on CVD prevention are essential. Additionally, research should explore the long-term effects of AC on various health outcomes, not just QoL, in order to better understand the comprehensive benefits of AC. Additionally, policymakers and urban planners must prioritize infrastructural improvements, such as safer roads and extended bike networks, to facilitate AC practice. Financial incentives and public health campaigns could further promote AC, potentially contributing to significant public health advantages and ecological benefits by reducing CO_2_ emissions.

## 5. Conclusions

This systematic review shows that daily AC, such as walking and cycling, significantly reduces CVD risk. Despite some methodological variability and biases in the included studies, the certainty of evidence strongly supports AC as a preventive measure for CVD. Future research should standardize AC definitions and methodologies to better understand its impact. Health professionals and policymakers should promote AC through infrastructure improvements and policies, as it not only enhances public health but also contributes to environmental sustainability by reducing CO_2_ emissions.

## Figures and Tables

**Table 1:** Primary and secondary search terms.

Primary Terms	Secondary Terms
Commuting Cardiovascular Diseases	CVD Active travel Active transportation Active transport AC Commuter Walking to work Walking Transportation Cycling to work Cycling Bicycling Commuter cycling Cardiovascular incidence Cardiovascular mortality Cardiovascular event CHD Cerebrovascular disease Rheumatic heart disease Rheumatic heart diseases Myocardial infarction/epidemiology Cardiovascular diseases/epidemiology Stroke Heart disease Blood vessel Prevention

CVD: cardiovascular diseases; AC: active commuting; CHD: coronary heart disease.

**Table 2:** Methodological quality evaluation of included studies using the National Institute of Health (NIH) quality assessment tool for observational cohort and cross-sectional studies.

First Author (Year)	A	B	C	D	E	F	G	H	I	J	K	L	M	N	O
Bauman et al., (2017) [43]	1	1	1	0	1	1	1	1	1	1	1	X	0	1	Good
Blond et al., (2016) [44]	1	1	1	1	1	1	1	1	1	1	1	X	0	1	Good
Eriksson et al., (2020) [45]	1	1	X	1	1	1	1	1	1	1	1	X	1	1	Good
Fan et al., (2019) [46]	1	1	0	0	1	1	1	1	1	1	1	1	1	1	Good
Loprinzi & Davis, (2015) [47]	1	1	1	1	1	1	1	1	1	1	1	X	1	1	Good

A: Was the research question or objective in this paper clearly stated? B: Was the study population clearly specified and defined? C: Was the participation rate of eligible people at least 50%? D: Were all subjects selected or recruited from the same or similar population? Were inclusion and exclusion criteria for being in the study prespecified and applied uniformly to all participants? E: Was the sample size justification, power description, or variance and effect estimates provided? F: For the analysis in this paper, were the exposure(s) of interest measured prior to the outcome(s) being measured? G: Was the timeframe sufficient so that one could reasonably expect to see an association between exposure and outcome if it existed? H: For exposures that can vary in amount or level, did the study examine different levels of the exposure as related to the outcome (e.g., categories of exposure, or exposure measured as continuous variables)? I: Were the exposure measures (independent variables) clearly defined, valid, reliable, and implemented consistently across all study participants? J: Was the exposure(s) assessed more than once over time? K: Were the outcome measures (dependent variables) clearly, valid, reliable, and implemented consistently across all the study participants? L: Were the outcome assessors blinded to the exposure status of participants? M: Was loss to follow-up after baseline 20% or less? N: Were key potential confounding variables measured and adjusted statistically for their impact on the relationship between exposure(s) and outcome(s)? O: Quality rating (Good, Fair, Poor); 0: No; 1: Yes; X: Cannot determine/Not applicable/Not reported.

**Table 3:** Data extraction of included articles.

Study Author	Country	Date Source	Study Design	Sample Size (n)	Age Range (Years)	Follow Up (Years)	Exposure	Exposure Measurement	Other PA Domains	Analysis Method	Outcome Definition	Events (n)	Controlling or Adjustment Variables
Bauman et al., (2017) [43]	Denmark	Danish MONICA study	Cross-sectional	2.829	30–61	19	Walking, bicycling to work	Self-reported	Leisure time PA, sport participation	Cox proportional hazards regression	All-cause mortality	668/2829	Age, sex, BMI, educational level, occupation, housing, waist and hip circumference
											CHD mortality	290/2829	
											CVD mortality	125/2829	
Blond et al., (2016) [44]	Denmark	Diet, Cancer and Health study	Prospective Cohort	53.723	50–65	20	bicycling to work	Self-reported	Leisure time cycling, and other PA	Cox proportional hazards regression	CHD event	2892/53.723	BMI, educational level, hypertension medication, hypercholesterolemia medication, self-reported diabetes medication, diet, alcohol, smoking
Eriksson et al., (2020) [45]	Sweden	Health Profile Institute	Prospective Cohort	318.309	18–74	17	Walking, bicycling, public transportation or car	Self-reported	Leisure time exercise, physical work situation	Cox proportional hazards regression	CVD events (fatal or non-fatal myocardial infarction, angina pectoris, or ischemic stroke)	5714/318.309	Age, sex, BMI, VO2max, beta-blockers, educational level, diet, smoking, perceived overall health
Fan et al., (2019) [46]	China	CKB data	Prospective cohort	104.170	35–74	10	Walking, bicycling	Self-reported	Leisure sedentary time, occupational commuting household	Cox proportional hazards regression	Ischemic heart disease	5374/104.170	Age, sex, BMI, educational level, marital status, household income, occupation, alcohol, smoking, red meat intake, fresh fruits and vegetables intake, hypertension, diabetes mellitus, family histories of heart attack or stroke
											Ischemic stroke	664/104.170	
											Hemorrhagic stroke	4834/104.170	
Loprinzi & Davis, (2015) [47]	U.S.A.	NHANES cycles	Prospective cohort	12.321	20–85	5	Walking, bicycling to work	Self-reported	Moderate-to-vigorous intensity aerobic PA, muscular strength activities	Cox proportional hazards regression	All-cause mortality	654/12.321	Age, sex, BMI, ethnicity, educational level, smoking, C-reactive protein
											CVD mortality	231/12.321	

BMI: body mass index; CHD: coronary heart disease; CVD: cardiovascular diseases.

**Table 4:** The GRADE certainty of evidence rating summary of findings of observational research.

Number of Studies (Subjects)	Risk of Bias	Inconsistency	Indirectness	Imprecision	Publication Bias	Grades of Recommendation
5 (*n* = 491,352)	Low	Low	High	High	High	Strong

The GRADE system defines four levels of evidence (high, moderate, low and very low) and two recommendation levels (strong or weak).
